# Liver-Stage Specific Response among Endemic Populations: Diet and Immunity

**DOI:** 10.3389/fimmu.2015.00125

**Published:** 2015-03-20

**Authors:** Sarat Kumar Dalai, Naveen Yadav, Manoj Patidar, Hardik Patel, Agam Prasad Singh

**Affiliations:** ^1^Institute of Science, Nirma University, Ahmedabad, India; ^2^Infectious Diseases Laboratory, National Institute of Immunology, New Delhi, India

**Keywords:** *Plasmodia*, liver-stage immunity, natural habit, sterile protection, chloroquine and chemoprophylaxis

## Abstract

Developing effective anti-malarial vaccine has been a challenge for long. Various factors including complex life cycle of parasite and lack of knowledge of stage specific critical antigens are some of the reasons. Moreover, inadequate understanding of the immune responses vis-à-vis sterile protection induced naturally by *Plasmodia* infection has further compounded the problem. It has been shown that people living in endemic areas take years to develop protective immunity to blood stage infection. But hardly anyone believes that immunity to liver-stage infection could be developed. Various experimental model studies using attenuated parasite suggest that liver-stage immunity might exist among endemic populations. This could be induced because of the attenuation of parasite in liver by various compounds present in the diet of endemic populations.

## Introduction

**Figure 1:**
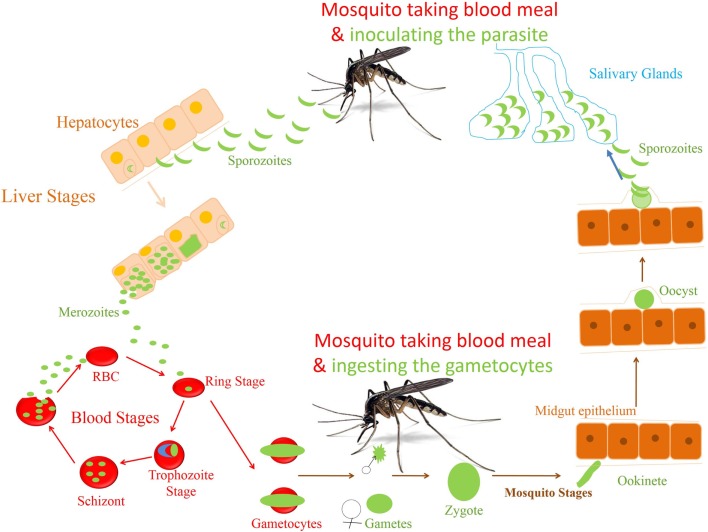
**Life cycle of *Plasmodium falciparum***. The life cycle of parasite *P. falciparum* starts in human with inoculation of parasite through mosquito. From the site of injection, sporozoite (SPZ) reaches to the liver and infect hepatocytes. The SPZ multiplies and produces thousands of blood stage infective merozoites. These merozoites enter the blood stream and infect the RBCs to start the erythrocytic cycle of parasite. In RBC, they go through different stages of development before they release the merozoites to infect new RBCs. A small percentage of asexual parasite transforms into sexual form, i.e., gametocytes, which finally develop into sporozoites in the mosquitoes.

Malaria along with HIV and TB poses great challenge to human health. More than 200 million people are at high risk and millions are dying (particularly, children) every year across the globe (1). Although anti-malarial drugs have helped bring down the severity and mortality of malaria in endemic regions, emergence of drug resistant parasite poses a great challenge to the human health prompting the urgent need for vaccine(s). Developing effective vaccines, however, has been challenging because of the complex life cycle of *Plasmodia*, which starts with the asymptomatic liver stage followed by the symptomatic blood stage infection (Figure 1). Various studies have shown the generation of protective immunity against the blood stage infection after repeated exposure to the parasite (2), but it is questionable against the liver stage (3, 4). However, experiments, using radiation or genetically attenuated sporozoite (RAS or GAS) that fails to complete their developmental cycle in liver, demonstrate the induction of sterile immunity in rodents and humans (2, 5–7). Even chemoprophylaxis and sporozoite (CPS) immunization that kills the parasite at early stage in RBCs (restricting its development to the liver) has shown similar results (2, 5, 7, 8). Although humoral and cell mediated responses are required to develop protection, CD8^+^ T cell response, generated by attenuated parasite, seems to play a critical role in providing protracted protection at liver stage (2, 3, 5–8). These findings prompted us to think that there is possibility of inducing liver-stage specific immune responses in humans by the parasite that might be attenuated or restricted to liver during natural infection.

People living in different subcontinents with high endemicity for malaria have been shown to have varying degree of susceptibility to infection (Table 1) (1). It becomes difficult to protect the host if the parasite enters the blood stage without being interrupted at liver stage as parasite load in blood could be uncontrollably high. Therefore, restricting the parasite to the liver could generate the protective immunity against malaria indicating the differential susceptibility of endemic people to challenge. Food habits have been shown to have a major impact on the health and modulation of immune response. For example, in India, people consume many herbs/spices as a part of their daily diet, which has been shown to have an anti-malarial activity, as explained later. This article has made an attempt to explain how parasites could be attenuated or restricted “naturally” to either liver stage or blood stage by the diet of people living in malaria endemic areas, potentially helping generate liver-stage specific immune responses.

**Table 1:** **List of countries with their Union Territory population, malaria endemic population, malaria confirmed cases, percent population at high risk, and percent incidence of malaria in 2011**.

Country	(A) UN population	(B) Population at high risk	(C) Population at high risk (%) (B × 100)/A	(D) Malaria confirmed cases	(E) Incidence of malaria (%) (D × 100)/B
**China**	**1,347,565,324**	**191,908**	** 0.014**	**4498**	**2.34**
**Brazil**	**196,655,014**	**4,523,065**	**2.3**	**267,045**	**5.9**
Nepal	30,485,798	1,127,975	3.7	71,752	6.36
South Africa	50,459,978	2,018,399	4	9866	0.49
Thailand	69,518,555	5,561,484	8	24,897	0.45
**India**	**1,241,491,960**	**273,128,231**	**22**	**1,310,367**	**0.48**
**Madagascar**	**21,315,135**	**6,394,541**	**30**	**224,498**	**3.51**
Myanmar	48,336,763	17,884,602	37	567,452	3.17
Timor-Leste	1,153,834	888,452	76.99	36,064	4.06
Mali	15,839,538	14,255,584	89.99	1,293,547	9.07
Papua New Guinea	7,013,829	6,592,999	94	1,025,082	15.55
Vanuatu	245,619	243,163	99	5764	2.37
Solomon Islands	552,267	546,744	99	80,859	14.79
Nigeria	162,470,737	162,470,737	100	3,392,234	2.09
Angola	19,618,432	19,618,432	100	2,534,549	12.92
Ghana	24,965,816	24,965,816	100	3,240,791	12.98
Zambia	13,474,959	13,474,959	100	4,607,908	34.19

## Challenges in Malaria Vaccine Development

Sterile protection to malaria even in endemic populations is not yet fully understood. Even vaccination has failed to induce the desired protection because of involvement of many complex factors (9–16). However, researchers have designed strategies that could provide sterile protection. Both blood and liver vaccination strategies are under different phases of clinical trials (17). The majority of the efforts to understand the immune response against *Plasmodia* infection in humans are directed against blood stage infection. But none of the blood stage vaccine candidates that have been tried so far demonstrated appreciable efficacy (18, 19). The same is also true for the liver-stage subunit vaccine candidates (20). Moreover, unlike the rationale of choosing blood stage vaccine candidates, the antigens for liver-stage vaccines have not been selected based on the understanding of protective immune response in humans against liver-stage infection, a probable reason for not having the right liver stage antigenic target. RAS, GAS, and CPS have shown appreciable efficacy (2, 5–8). Despite promising results, these approaches might not be feasible to adopt for mass vaccination. Considering the world population at risk of malaria, the feasibility of making billions of doses and maintaining the quality are very challenging. Second, GAS has been shown to revert to infectious parasite (21, 22) posing a threat to people expected to take vaccine for prophylaxis. Although CPS immunization is an attractive vaccination approach, drug resistant parasites and side effects of drug possess challenges. Therefore, approaches like a subunit vaccine will be good alternative. Inducing sterile protection by subunit vaccines requires identification of the right antigenic targets. From the experience of LSA-1 as vaccine candidate, it is critical to have new targets that would induce humoral and T cell responses. Unless we understand the nature of immune response vis-à-vis protection that exists in endemic population, it will be difficult to identify the targets. Because of the lack of knowledge of liver-stage specific immune responses among endemic populations, it is strongly believed that protective immunity to liver stage does not exist; hence efforts to make a liver-stage vaccine have not been prioritized, a reason in our opinion, for not having an effective vaccine against malaria even decades after the trial of RAS vaccination.

## Existence of Natural Immunity to Liver Stage

Many factors including genetic diversity, environmental conditions, and mosquito species do contribute to the differential susceptibility to infection (23–25). While this is true for most of the people living in different parts of the world, we strongly feel that the gain of such differential protection could have a direct correlation with the ability of endemic populations to generate liver-stage specific immune responses. Even though there is blood stage specific protective immunity in people in malaria endemic areas (2), often they come down with the infection (3). It indicates that protective immunity against the blood stage might not be sufficient, and therefore, liver-stage immunity is required to eliminate the parasite. Experiments suggest that achieving protective immunity requires both CD8^+^ T cells and antibody response (26) because CD8^+^ T cells are essential for liver-stage parasite while humoral response is key to control blood stage (27). Although fewer studies have been done in endemic populations, there is clear indication that immune responses to liver stage leading to protection could be achievable. In support of this, the studies conducted by the research groups of Marc Connelly and Adrian Hill in Africa have shown that protection, although among a minute endemic population, correlates with immune response to liver-stage antigen (LSA) (23, 28, 29). As discussed before, immunization with attenuated or drug restricted *Plasmodium falciparum* sporozoite is very effective in both animal models and in humans. Results indicate that protection is achievable either by vaccinating the host or exposing the host to the parasite attenuated or restricted naturally.

## Endemicity vs. Infectivity among Endemic Populations

**Figure 2:**
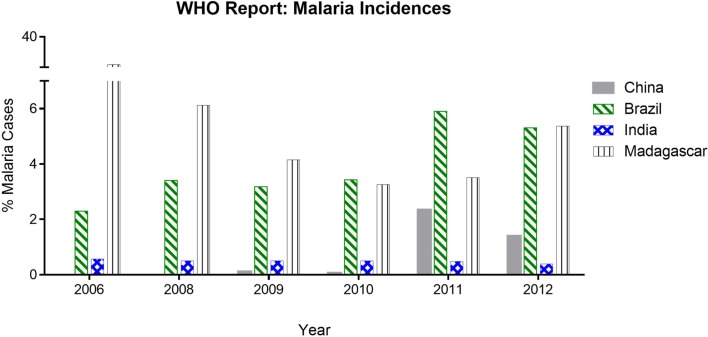
**Malaria incidences based on WHO report analysis**. The data show the % of malaria cases (in China, Brazil, India, and Madagascar) during 2006–2012 reported in World Malaria Report. India and Madagascar have comparative high endemicity but varying infectivity whereas China and Brazil having low endemicity but proportionally higher infectivity.

Our analysis of WHO data shows that people of certain subcontinents are less susceptible to malaria than others having similar risk of high endemicity (Table 1). People living in Indian subcontinents have been showing lower incidence of malaria compared to other countries having endemicity either lower or higher than India. India is having 0.48% malaria incidence, which is seven times lower than that of Madagascar, i.e., 3.50%. Malarial endemicity of India is 22% while that of Madagascar is 30% (Table 1). Various states of India, e.g., Arunachal Pradesh, Meghalaya, Mizoram, Tripura, Jharkhand, and Odisha are having high risk (72–100%) of infection (Table 2) (30), but the rate of malaria infection is about 0.72%. In contrast, China and Brazil where only 0.014–2.3% population at high risk but incidence of malaria is 2.3–5.9%, which is threefold to eightfold higher than that of India (Table 1), based on the data of 2011. Further analysis of data from 2008 to 2012 suggests that the incidence of malaria remain consistently low even if the high risk population has not decreased significantly as compared to countries like China and Brazil (Figure 2). It is possible that people living in specific regions of high endemic zones of African countries or Brazil might have the similar trends of malaria incidence. It is known that immune responses against blood stage infection are the major factor in providing protective response. However, it is possible that the low incidence of Malaria among endemic populations like in India could be due to the contribution of the liver-stage immune responses generated among them. This might be because of their unique diets playing an important role in attenuating the parasite, a critical factor in generating liver-stage specific response.

**Table 2:** **List of states of India with their population, malaria endemic population, malaria confirmed cases, percent population at high risk, and percent incidence of malaria**.

State	(A) Population	(B) Population at high risk	(C) Population at high risk (%), (B × 100)/A	(D) Malaria confirmed cases	(E) Incidence of malaria (%), (D × 100)/B
Mizoram	1,091,014	1,091,014	100	8861	0.81
Tripura	3,671,032	3,671,032	100	14,417	0.39
Arunachal Pradesh	1,382,611	1,257,586	91	13,950	1.11
Jharkhand	32,966,238	28,791,697	87	160,653	0.56
Odisha	41,947,358	36,494,063	87	308,968	0.85
Meghalaya	2,964,007	2,139,948	72	25,143	1.18
India	1,210,569,573	26,63,25,306	22	1,310,656	0.49

## Promoting Liver-Stage Immune Responses by Naturally Attenuated Parasite

Based on our understanding from various reports, it is possible that many compounds derived from the herbs/spices could attenuate the parasite provoking immune responses to malaria. People, especially those living in the continents of Asia and Africa consume herbs and spices as part of their daily diet that have many medicinal effects; e.g., turmeric, garlic, and black pepper are consumed daily by Indians in almost all food items (31–34). The compounds from various sources listed in Table 3 are shown to act on blood or liver stage parasite. It is possible that some of those compounds help induce liver-stage specific immune responses.

**Table 3:** **The compounds derived from various sources with their anti-malarial activity, mode of action and estimated concentration**.

Fruits or herbs	Compound present	Estimated concentration	Mode of action (anti-malarial activity)	Reference
Apples, oranges, lemons, onions, nuts, garlic, neem leaves	Quercetine (flavonoid) 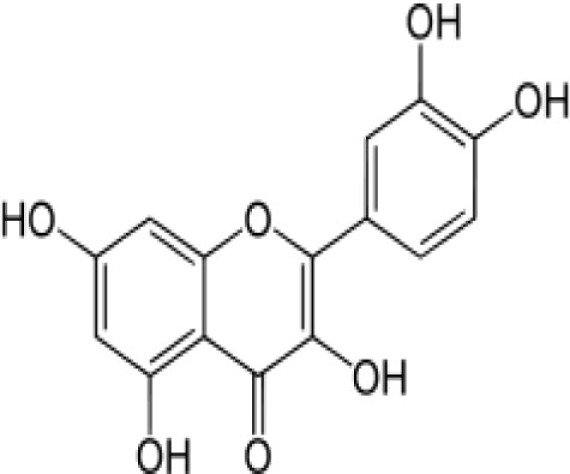	32 mg/100 g of red onion; Daily intake 12.9 g/day)	Inhibition of heme polymerization by chelating free available hemin for polymerization	(35)

Grapefruit, lime, pomegranate, parsley	Quinine (alkaloids) 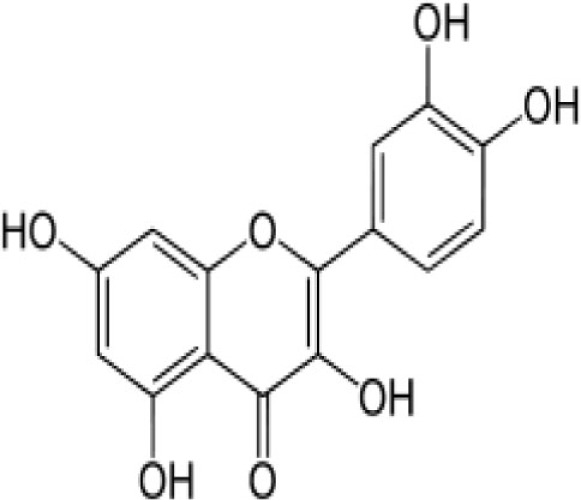	~100 mg total alkaloids, including quinine in a cup of traditional quinine bark tea	Blocks malaria from reproducing by binding to the parasite’s DNA Inhibition of hemozoin bio-crystallization, which facilitates the aggregation of cytotoxic heme. Free cytotoxic heme accumulates in the parasites, causing their deaths.	(36, 37)

Strawberry, pomegranates and the best source, red raspberry seeds/red raspberries	Ellagic acid (polyphenol) 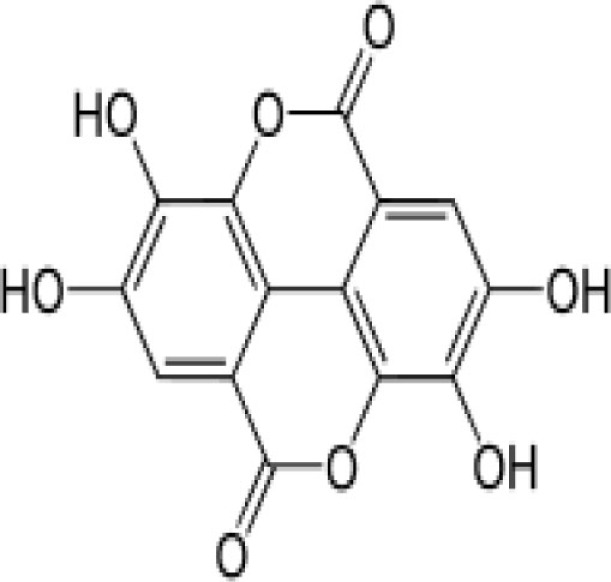	50.06 mg/10 gm of strawberry	Inhibition of β-hematin (hemozoin) formation Act on trophozoite and early schizont forms of the parasites. This erythrocytic stage of the malaria life cycle is the most metabolically active phase, with protein, RNA, and DNA synthesis taking place.	(38, 39)

Tomatoes, carrots, pears, coconut, leek, onion, spinach, broccoli, avocado, eggplant, mango, apples, apricot, banana, radish, turmeric, echinacea tea, marshmallow root	Arabinogalactan (polysaccharides) 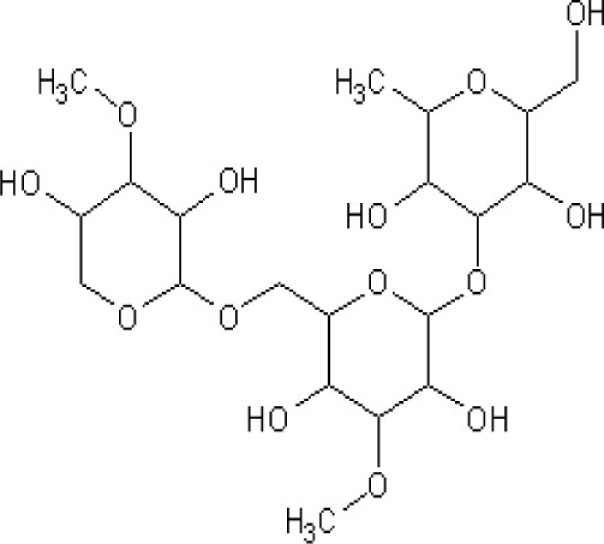	15–25% in larch	Macrophage activator Support the monocyte production	(40, 41)

Basil oil	Quinones 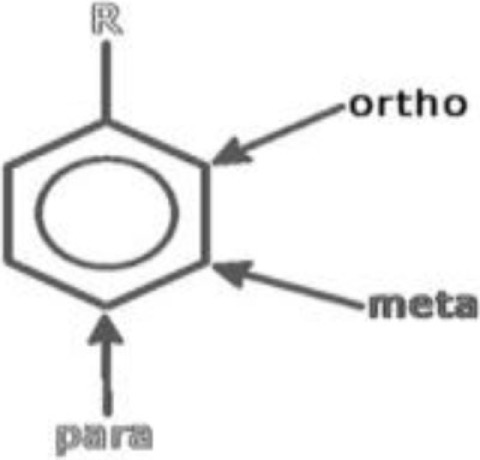	N/A	Inhibition of parasite mitochondrial electron transport chain and respiratory chain without affecting the host mitochondrial system	(42, 43)

Turmeric	Curcumin (curcuminoid) (natural phenols) 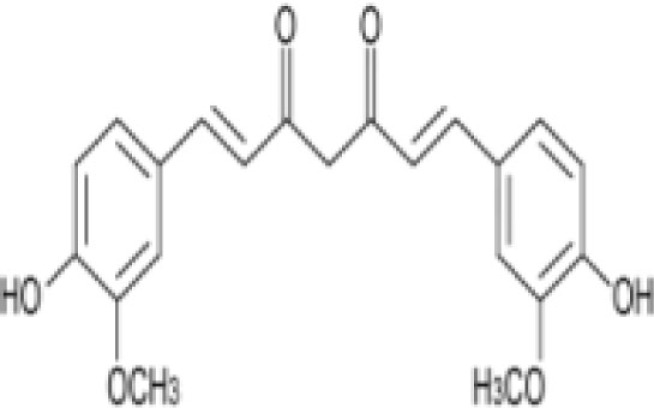	3.14% by weight in pure turmeric powder; Alleppey turmeric: 4–7% curcumin; Madras type: 2% curcumin	Anti-oxidant activity Curcumin induced generation of ROS may lead to histone hypoacetylation and DNA damage that account for the parasiticidal effect of curcumin	(34, 44–51)

Black pepper	Piperine 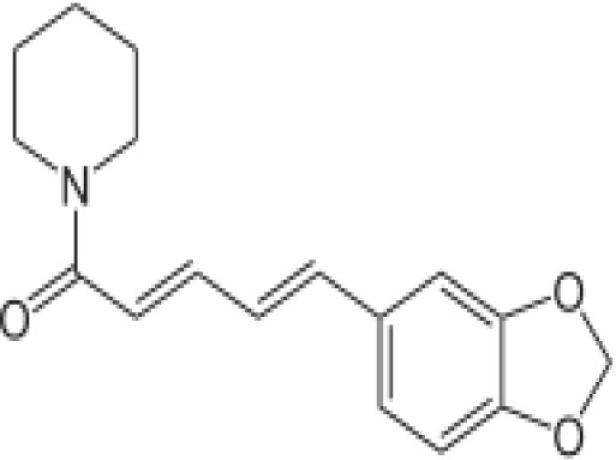	5–10%	Enhances the bioavailability of curcumin by 2000-fold	(33)

Cinnamon	Cinnamic acid derivatives 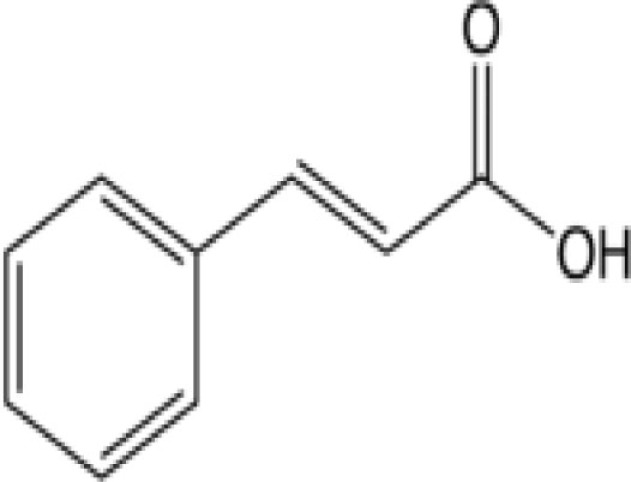	0.96–2.91%; 0.87 mg/g	Inhibit the transport of monocarboxylate across erythrocyte and mitochondrial membranes Inhibit parasite growth and they are equally effective at the young (ring) and the mature (trophozoite) stages of parasite development	(52, 53)

Garlic cloves	Allicin, organosulfur compound 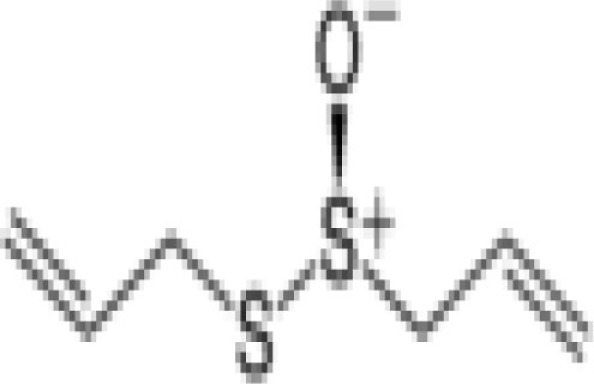	1–3% (2.8–7.7 mg/g found in Romanian red)	Inhibits circumsporozoite protein processing and prevents sporozoite invasion of host cells *in vitro*. *In vivo* mice injected with allicin had decreased *Plasmodium* infections compared to controls When sporozoites were treated with allicin before injection into mice, malaria infection was completely prevented Immunomodulatory activities (preferentially enhances pro-inflammatory immune responses)	(54–56)

Fenugreek	In leaves: alkaloids, saponin, tannin like phenolic compounds, flavonoids and steroids 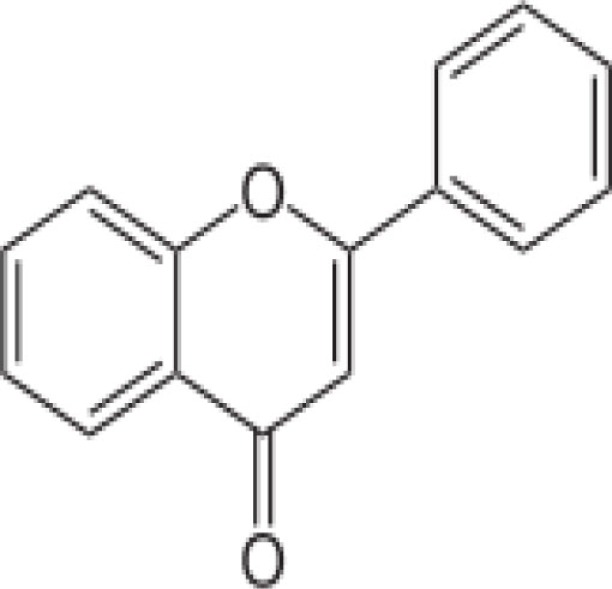	Fenugreek contains 35% alkaloids and 4.8% saponin	Hemozoin inhibitors The alkaloidal, ethanol, and butanol extract of fenugreek has been documented to possess anti-plasmodial activity against *in vitro* culture of chloroquine sensitive and resistant *Plasmodium falciparum* Presence of flavonoids and polyphenols has been found to be responsible for powerful anti-oxidant activity Fenugreek seeds also have capacity to increase the immunity power and to fight against the parasites	(57, 58)

Peanuts, grapes, grape juice, berries, e.g., blueberries and black berries	Resveratrol (stilbenoid, a type of natural phenol) 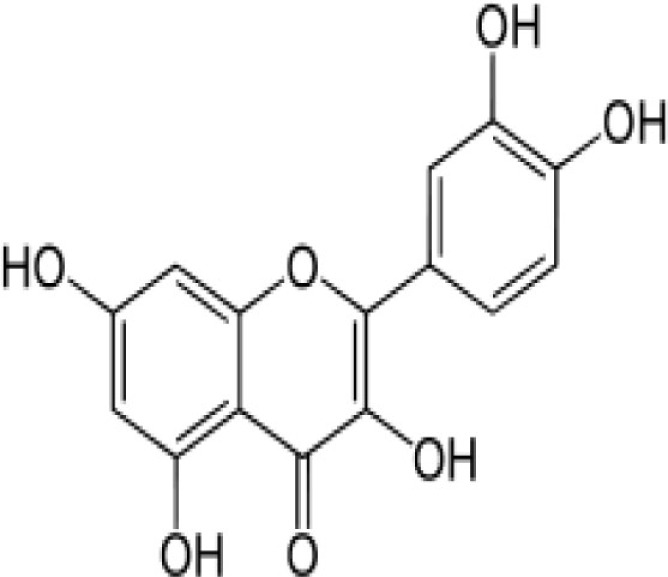	0.01–0.26 mg in peanuts	Treatment of parasite-infected red blood cells with resveratrol significantly reduces their ability to adhere to the body’s cells lining small blood vessels. That reduction in binding to blood vessels is predicted to greatly lessen the probability of developing severe clinical manifestations of malaria, according to the study.	(59)

Ginger	N/A	N/A	Nausea and vomiting are also common symptoms of malaria, which may explain the widespread use of ginger as one component of traditional remedies for malaria It stimulates production of the main anti-oxidant enzyme glutathione peroxidase, this detoxification-related enzyme improves the liver function and binds toxins. The compounds of ginger inhibit the malaria parasite.	(60)

Cold-pressed coconut oil, fresh and dried coconut, coconut milk, bitter melon	Lauric acid (Saturated fatty acid) 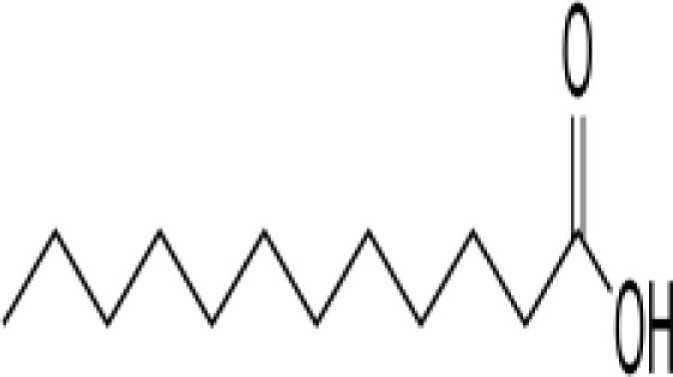 ferulic acid and *p*-coumaric acid	Pure coconut oil contains about 50% lauric acid	When lauric acid is converted into monolaurin, a monoglyceride compound, which exhibits antiviral, antimicrobial, anti-protozoal, and anti-fungal properties. It acts by disrupting the lipid membranes in organisms like fungus, bacteria, and viruses, thus destroying them Coconut has anti-oxidant compounds	(61, 62)

Quinine, arabinogalactan, curcumin, piperine, ellagic acid, quercetin, alkaloids, flavonoids, cinnamic acid derivatives, and allicin to name a few compounds are known to have anti-malarial activity, present in the diet. Many anti-malarial drugs target heme polymerization, essential for parasite to complete its life cycle. Quercetine (apples, oranges, lemons, onions, nuts, garlic, and neem leaves) is known to act on the blood stage parasite by preventing heme polymerization through sequestration of free hemin by forming quercetin–hemin complex (e.g., against *P. falciparum* 3D7) (35). It has also shown to inhibit parasite growth in a dose dependent manner by interfering in the permeability pathways. Quinine (grapefruit, lime, pomegranate, and parsley) also prevents heme polymerization as well as binds to the DNA of parasite at schizont stage and blocks its reproduction (36, 37). Ellagic acid (strawberry, pomegranates and the best source, red raspberry seeds/red Raspberries) has been shown to act on mature trophozoite and young schizont of blood stage parasite while inhibiting hemozoin formation (38, 39, 63). It has also been shown that ellagic acid potentiates the activity of anti-malarial drugs like chloroquine, mefloquine, artesunate, and atovaquone. According to a curative test, the ED_50_ of ellagic acid for *Plasmodium vinckeipetteri* was around 1 mg/kg/day by the intraperitoneal route. Under the same conditions, artesunate, the most effective semi-synthetic derivative of artemisinin, shows an inferior ED_50_ of 5 mg/kg/day. Several studies treating mice intra-peritoneally with ellagic acid before parasite inoculation showed high-level reduction (between 79 and 93%) of parasitemia by day 6 post-infection suggesting a prophylactic effect of ellagic acid (38, 39, 63).

Arabinogalactan (tomatoes, carrots, pears, coconut, leek, onion, spinach, broccoli, avocado, eggplant, mango, apples, apricot, banana, radish, turmeric, echinacea tea, and marshmallow root) enhances monocytes production and also activates macrophages that play an important role in phagocytosis of the parasite, thus lowering the parasite load, and presenting parasite antigens to help potentiate the liver-stage specific T cell responses (40, 41). Quinones (basil oil) inhibit the parasite development at schizont stage by blocking its mitochondrial electron transport and respiratory chain without affecting the host (42, 43).

Curcumin (turmeric) acts by damaging the parasite DNA and altering the histone acetylation that accounts for the parasiticidal activity on blood stage parasite (43–48). It has been shown that curcumin inhibits chloroquine-resistant *P. falciparum* growth in a dose dependent manner with an IC_50_ of 5 μM (46). Furthermore, oral administration of curcumin to mice infected with *Plasmodium berghei* reduces blood parasitemia by 80–90% and enhances the survival. Padmanaban et al. evaluated the *in vivo* efficacy of the curcumin–artemisinin combination (46). The results indicated that a, J3-arteether or curcumin monotherapy at the indicated doses prolongs the survival of *P. berghei*-infected mice but does not confer complete protection. However, combining curcumin with a, J3-arteether treatment reduces the infectivity further resulting 100% protection. Consumption of black pepper, source of piperine, is very common even among non-endemic populations. Piperine has been shown to enhance the bioavailability of curcumin by 2000-fold (33). The amount of curcumin consumed by endemic population (e.g., India) appears to support the fact that it would be enough to attenuate the parasite in the liver (34, 49–51). Cinnamic acid (cinnamon) derivatives have been shown to act at the young (ring) and the mature (trophozoite) stages of parasite development by inhibition of lactate transport or of mitochondrial respiration required for energy generation (52, 53, 64).

Allicin (garlic cloves) has been shown to prevent the parasite invasion in hepatocytes, reduce parasite load in blood, and enhance survival. It has been shown that the circumsporozoite protein (CSP) of Plasmodium sporozoites is proteolytically processed by a parasite-derived cysteine protease, and this processing event is associated with sporozoite invasion of host cells (54). It is found that 10, 25, or 50 μm allicin inhibit CSP cleavage, which is comparable to that observed with 10 μm E-64, a cysteine protease inhibitor that inhibits CSP processing and prevents invasion of host cells *in vitro* and *in vivo*. Mice injected with allicin showed reduced parasitemia compared to controls. Furthermore, treatment of sporozoite with allicin before injecting into mice completely prevented malaria infection suggesting allicin might directly attenuate the parasite (55). The protective effect of allicin seems to be influenced by improved host immune responses. It has been demonstrated in a rodent malaria model of *Plasmodium yoelli* (17XL) infection that allicin treatment enhances the production of pro-inflammatory mediators like IFN-γ, TNF, IL-12p70, and NO. The numbers of CD4+ T cells, DCs, and macrophages were significantly higher in allicin-treated mice. Allicin also promoted the maturation of CD11c+ DCs, while it did not cause major changes in IL-4 and the level of anti-inflammatory cytokine IL-10 (54–56).

Fenugreek is a common household item in special food preparation of Indians. Alkaloids and flavonoids derived from Fenugreek have been shown to inhibit the hemozoin formation and possess anti-plasmodial activity against chloroquine sensitive and resistant *P. falciparum*. It was found that fenugreek extract in a dose of 50, 100, or 250 mg/kg showed immunomodulatory property through various mechanisms including weight of thymus, delayed type of hypersensitivity response, humoral immunity and phagocytosis (57, 58, 65, 66). Peanuts, grapes, grape juice, and berries are common food consumed all over the world. Resveratrol, derived from the same have been shown to significantly reduce the ability of infected RBCs to adhere to the body’s cells lining small blood vessels. Such phenomenon is predicted to greatly lessen the probability of developing severe clinical manifestations of malaria, according to the study (59).

**Figure 3:**
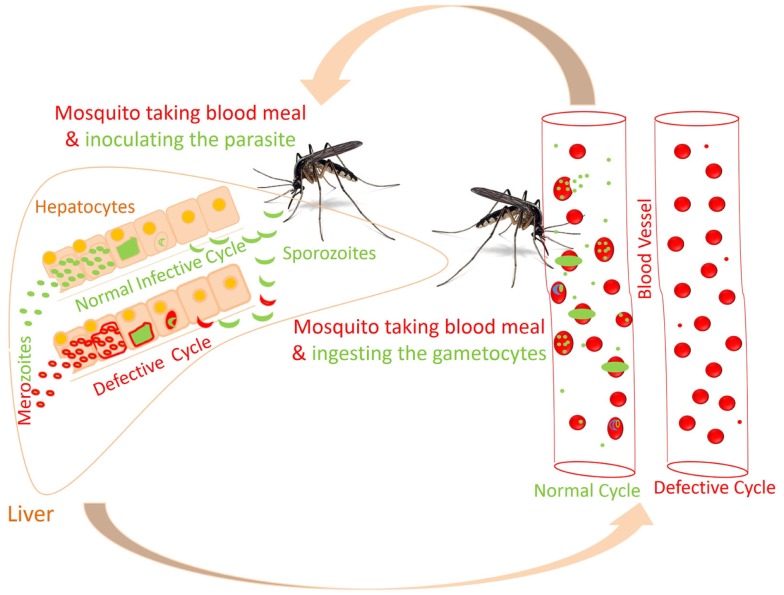
**Proposed altered life cycle of *Plasmodium* following consumption of diet containing anti-malarial compounds**. Action of various compounds derived from diet is depicted in the figure. Green colored parasite represents normal life cycle while that of red colored parasite represents defective life cycle because of action of compounds present in diet. Possibilities: SPZ not able to infect the hepatocytes; parasite development is interrupted at initial or late stage in hepatocytes; or merozoites released might not able to infect the RBCs. Certain compounds in diet might attenuate the parasite following infection in RBCs.

Frequent consumption or decoction of medicinal plants by the people in malaria endemic areas has been shown to help fight the infection. Evidence for this came from the longitudinal study undertaken by Foundation for Revitalization of Local Health Traditions (FRLHT) in endemic regions of Odisha, Andhra Pradesh, and Chhattisgarh of India. It showed that traditional plant decoction taken by people (tribal and non-tribal) reduced the incidence of malaria by more than threefold (in communication, 67–69). Based on the actions of said compounds present in diet and/or in decoction, it is possible that parasite development is largely interrupted at the blood stage; in other words the *Plasmodia* infection among endemic populations, as in case of India, would preferentially be restricted to the liver stage helping provoke immune response against the LSAs. Therefore, we hypothesize that natural diet of people of different subcontinent could attenuate *Plasmodia* at different stages of infection and thus help provoke the immune responses against malaria liver-stage infection enhancing protection (Figure 3). Recent findings from the studies of vaccination of host using infectious sporozoite under the cover of chloroquine support the hypothesis.

## Lessons from Chemoprophylaxis Study with Chloroquine and Determining Factors for Protective Immunity

**Figure 4:**
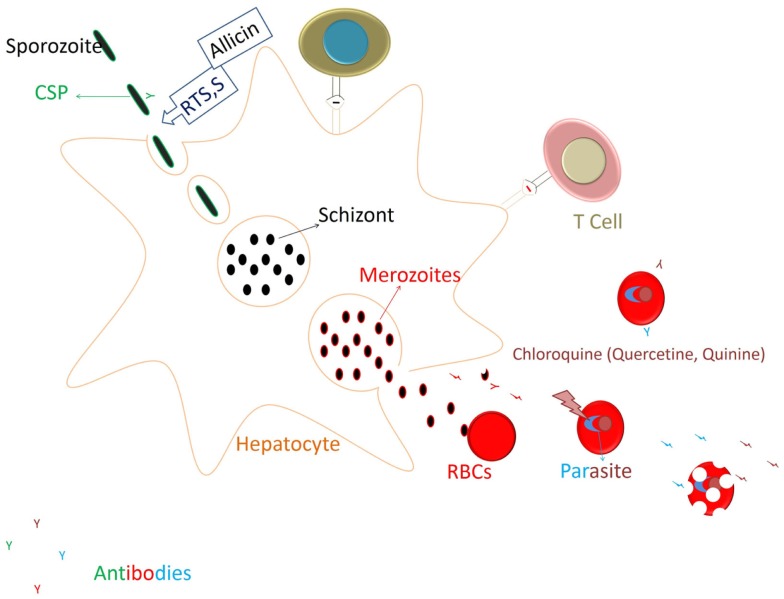
**Restricting the parasite development to Liver stage and generation of immune responses**. RTS,S vaccines targeting the circumsporozoite protein (CSP) of the parasite generates mainly antibody responses against the CSP that acts on the sporozoite to prevent its invasion into hepatocytes. Allicin from the garlic cloves also would act in similar manner (prevention of hepatocyte invasion by spz) by eliminating CSP processing. After invading the hepatocyte sporozoite will undergo further development, and merozoites released infect the RBCs and start degrading Hb. Heme released is polymerized to curtail its toxicity on the parasite. Chloroquine at this stage blocks the heme polymerization and kills the parasite. Thus, chloroquine (or compounds from food having similar action) would restrict the parasite development. Parasitic antigens released from the infected RBCs might generate the humoral and cellular responses to the blood stage parasite and also against the cross stage antigens from liver stage parasite (69).

In endemic areas, people are repeatedly exposed to malaria parasite and control the infection with immune responses directed against the blood stage, as supported by majority of the investigations (2), or with the help of anti-malarial drugs. It is possible that interruption of parasite development at different stages, either in liver or in blood, by natural means or drug usage favors generation of liver-stage specific immune responses (Figure 4) (70). This possibility is revealed by the CPS immunization studies in mice and humans. In CPS immunization, three doses of parasite under chloroquine cover induce sterile protection that correlates with CD8^+^ T cell responses directed against the pre-erythrocytic stage of parasite (7, 8). In these studies, the immunized volunteers have been shown to be protected for up to 2 years. Similarly, it is possible that compounds from dietary plants, as described above and medicinal decoction (during the diseased condition), would have similar actions like to Chloroquine. In fact, the effective anti-malarial drugs we use today are derived from the plants. In malaria endemic areas, though maternal antibodies help fight the infection during their early life; children are highly prone to infection, which might be due to their developing immune system. Their susceptibility to malaria infection increases further probably for the fact that consumption of herbs and spices as part of food is quite low or rare in children, which increases as they grow. Hence, with the advancement of age, repeated exposure to the parasite, and frequent consumption of the herbs and spices might help to develop the protective immunity against the malaria, which is further supported by the observation that after several years of exposure to parasite children do develop immunity to the severe life threatening malaria (2).

**Figure 5:**
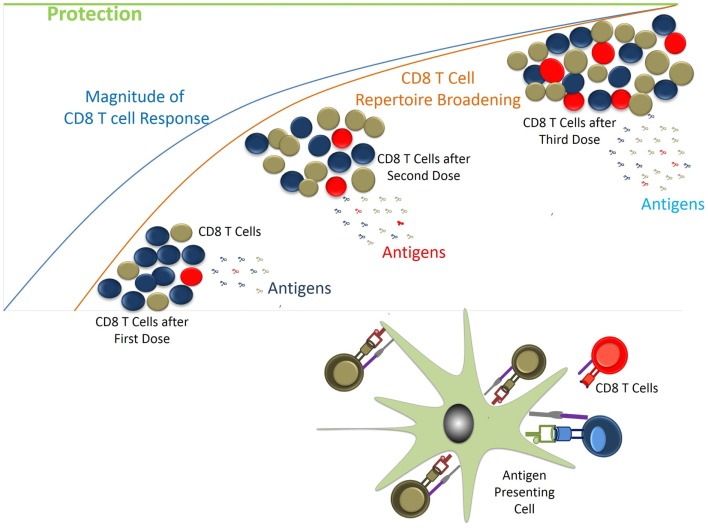
**Generation of CD8 T cell response vs. protection**. CD8 T cells are generated following exposure to parasite antigens through natural infection. Depending on the availability and affinity of antigens CD8 T cell clonotypes would have access to MHC/p complexes. Initially MHC/p in excess or with high affinity would preferentially induce the specific T cells. During subsequent antigen exposures other antigens would come into picture while response to previously generated CD8 T cells will be boosted. Gradually over time the desired repertoire of T cells with required frequencies would be generated helping protect the host from the incoming infection.

Although blood stage specific immune responses seem to be dominating protection, the presence of liver-stage specific immune responses should not be underestimated. Possible reasons for not having noticeable liver-stage immunity among endemic population could be the low antigen availability and the tolerogenic environment in liver that keeps the inflammation under control. Hence in case of malaria, the parasite load and its frequency of exposure are the key factors to develop protective immunity against liver-stage infection. In natural exposure, the parasite load appears to be lower in endemic population compared to the immunization regimen in experimental studies. However, there is possibility of induction and building up of liver-stage specific immune responses in endemic populations over the years of exposures contributing to the protection (Figure 5). Many pre-clinical vaccine studies including ours support the above notion (71–73). In CPS immunization studies, it has been seen that three doses of parasite (sporozoite) immunization under the cover of chloroquine through i.v. route within short time period induce the sterile protection (26). While two doses of 20,000 sporozoite (spz) immunization protect 40% of mice, three doses of the same protect 100% of mice following infectious sporozoite challenge. Similar results were also found in our experiments using attenuated parasite (γ-spz) immunization strategy (manuscript in preparation, 71). The higher level of protection correlates with the presence of higher number of multifunctional CD8^+^ T cells suggesting that multiple exposure to parasite antigens is required to achieve the protecting numbers of multifunctional CD8^+^ T cells (74). It is also possible that CD8^+^ T cell repertoire would be broadened for the subdominant antigens (antigens from various stages of development in liver) with repeated Ag exposure and ensue the protection against liver-stage malaria. Interestingly, it has been demonstrated that mice immunized with three doses of 10,000 spz under the cover of chloroquine were partially protected in contrast to mice that were given three doses of 20,000 spz (26). Further support came from the comparative studies in which the immunizations of attenuated parasites were done through intradermal (ID) vs. intravenous (IV) routes. ID route of immunization was thought to mimic the natural route of delivery of sporozoite through mosquito bite. It was found that immunization of mice through IV route was protective, while the same through ID route failed to protect the mice upon challenge (75). It has been demonstrated that parasite load in liver in case of ID immunization is very low compared to the IV (75) reflecting the low levels of the availability of antigens, which might be not sufficient to induce the desired magnitude of CD8^+^ T cells and to broaden the repertoire. To better understand the liver-stage specific immune responses, protected individuals in endemic areas should be included in large scale studies while CPS immunizations in humans could be taken as alternative positive control. Screening of protective antigens from those individuals will help the efforts of subunit vaccination.

## Conclusion

Over a decade ago, the malaria genome was sequenced, which has revealed that the parasite contains over 5000 genes. Researchers around the globe are actively engaged in identifying and characterizing the genes. We have not yet been very successful in making effective vaccines, which could generate the protective immune responses against various developmental stages of *Plasmodia* infection. It is well known that multiple immune mechanisms are required to prevent the infection, e.g., CD8^+^ T cells are critical for liver-stage infection as parasite remains inside the hepatocytes while humoral response is the key mechanism in blood stage infection where antibodies are required to prevent the free merozoites to infect the RBCs (27). In addition, to prevent the sporozoite from invading the hepatocytes, we require neutralizing antibodies. Hence, a vaccine should be made, which could generate a diverse immune response. RTS,S is the most successful subunit vaccine available that produces the neutralizing antibodies against the CSP, present on the surface of sporozoite. Currently, whole sporozoite vaccines (RAS or GAS) are also becoming popular as they confer the sterile protection at least in experimental models by activating CD8^+^ T cells. Here, RAS are mainly restricted to initial stage while GAS are designed to be restricted at early, mid, or late in liver-stage development. Interestingly, chemoprophylaxis studies with chloroquine have shown the activation of both CD8^+^ T cells and antibody response in which parasite is prevented at early blood stage (26).

Although we have understood mechanistically immune responses generated in animals or in experimental trials in humans, we have very little idea about the antigenic targets against which immune responses seems to be protective. And this is particularly true for liver-stage infection. Because there is lack of understanding of induction of protective immune response among endemic population against liver-stage infection, it has been very difficult to identify the targets. Therefore, systemic efforts must be made to understand liver-stage specific protective immune responses, particularly, in those populations living in malaria endemic area who are protected from the malaria by getting repeated exposure to the parasite. A recombinant protein of liver-stage parasite in adjuvant formulation will be an ideal formulation for mass scale immunization. A pre-erythrocytic stage vaccine should induce strong cellular immune response as well as develop long lasting immunological memory. New insight into parasite biology, stage specific expression profile, and characterization of carefully selected new antigens would provide new targets for interventions. Research institutes with focused R&D and skilled manpower must take the lead in the efforts to make vaccine against malaria by targeting antigens from multiple stages of *Plasmodia* infection.

## Conflict of Interest Statement

The authors declare that the research was conducted in the absence of any commercial or financial relationships that could be construed as a potential conflict of interest.
