# Corrigendum: Liver-Stage Specific Response among Endemic Populations: Diet and Immunity

**DOI:** 10.3389/fimmu.2015.00324

**Published:** 2015-06-22

**Authors:** Sarat Kumar Dalai, Naveen Yadav, Manoj Patidar, Hardik Patel, Agam Prasad Singh

**Affiliations:** ^1^Institute of Science, Nirma University, Ahmedabad, India; ^2^Infectious Diseases Laboratory, National Institute of Immunology, New Delhi, India

**Keywords:** plasmodia, liver-stage immunity, natural habit, sterile protection, chloroquine and chemoprophylaxis

In the original article on page 5 and 6 there are two errors in Table 3. The corrected figures are given in following table.

**Table:** 

Compound name	Incorrect figure	Correct figure	Reference
Resveratrol	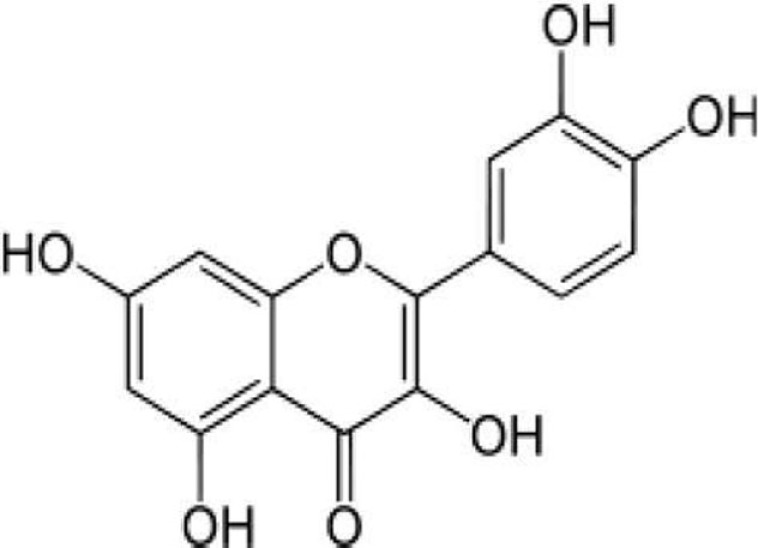	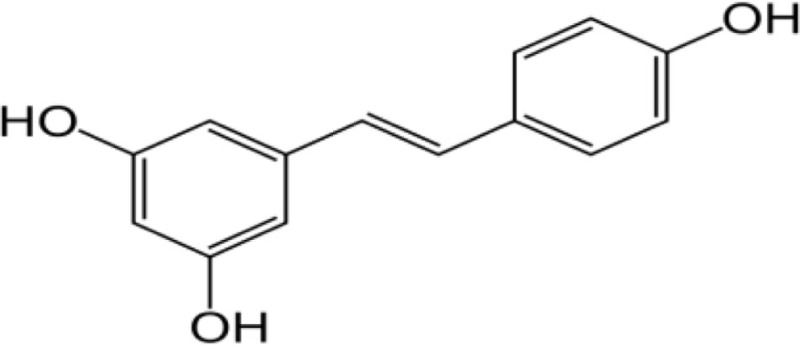	(1)
Quinine	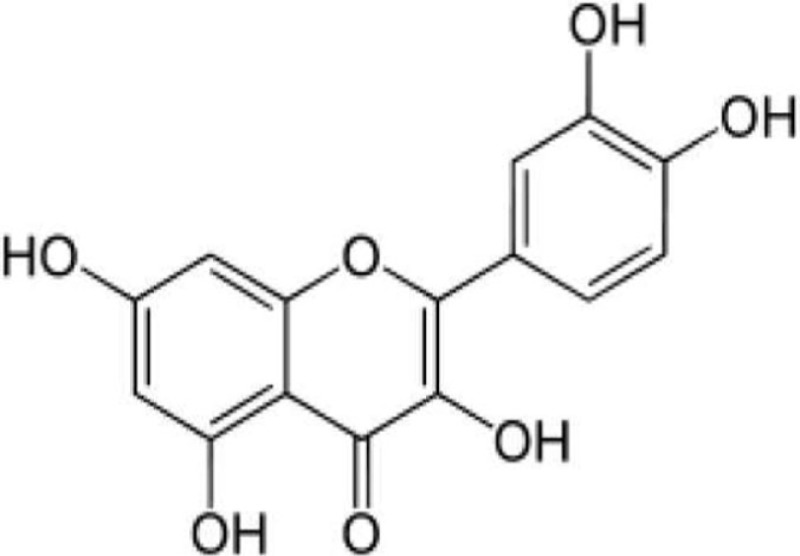	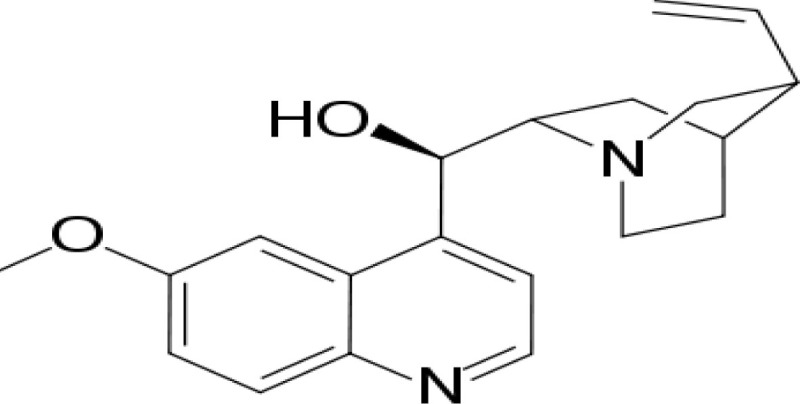	(2, 3)

## Conflict of Interest Statement

The authors declare that the research was conducted in the absence of any commercial or financial relationships that could be construed as a potential conflict of interest.
